# Factors associated with severe lung disease in an adult population with cystic fibrosis:a single-center experience

**DOI:** 10.3906/sag-1912-101

**Published:** 2020-06-23

**Authors:** Berrin ER, Ebru ÇELEBİOĞLU, Ebru YALÇIN, Deniz DOĞRU, Özlem ERDEN AKI, Ömrüm UZUN, Murat AKOVA, Uğur ÖZÇELİK, Nural KİPER, Salih EMRİ

**Affiliations:** 1 Medical Intensive Care Unit, Faculty of Medicine, Hacettepe University, Ankara Turkey; 2 Department of Chest Diseases, Faculty of Medicine, Hacettepe University, Ankara Turkey; 3 Pediatric Pulmonology, Faculty of Medicine, Hacettepe University, Ankara Turkey; 4 Department of Psychiatry, Faculty of Medicine, Hacettepe University, Ankara Turkey; 5 Department of Infectious Diseases and Clinical Microbiology, Faculty of Medicine, Hacettepe University, Ankara Turkey; 6 Department of Chest Diseases, Medicana Kadıköy, İstanbul Turkey

**Keywords:** Adult, cystic fibrosis, severe lung disease, risk factors

## Abstract

**Background/Aim:**

The patients with cystic fibrosis (CF) are living longer compared to the past, but respiratory failure is still the most common cause of mortality. The aim of this study is to investigate factors associated with severe lung disease in a cohort of adult patients with CF.

**Materials and methods:**

Demographic data, clinical and laboratory findings of the patients aged 18 years and more were collected and the patients were grouped according to forced expiratory volume in 1 s (FEV1) as severe group: < 40% and nonsevere ≥40%. Associations were investigated between groups and clinical outcomes.

**Results:**

A total of 76 patients were enrolled in the study. The mean age was 24.5 ± 5.25 years and 36 (47.4%) patients were female. In the severe group; the mean age was higher (27.1 ± 6.0 vs 23.6 ± 4.7, P = 0.013), the median Chrispin-Norman score of severe lung disease group was higher (14 (6–22) vs 5.5 (0–20), P< 0.001),hospitalization at least once in a year for intravenous antibiotic was more common (12/18 (66%) vs 19/58 (32%), P = 0.014). There was a positive correlation between body mass index (BMI) and lung function, indicating that lower nutritional status was related to lower FEV1, r2 = 0.21, P < 0.001. The median FEV1% was lower in patients with CF-related diabetes (38 (14–95) vs 66 (13–121), P = 0.042). Dornase alpha use and physiotherapy rate were higher in severe lung disease group (P = 0.008 and P < 0.001, respectively).

**Conclusion:**

Lower BMI, older age, presence of CF-related diabetes, higher radiologic scores, use of dornase alpha and physiotherapy and higher hospitalization rate for intravenous antibiotic therapy are significantly associated with severe lung disease.

## 1. Introduction

With advanced multidisciplinary care, nutritional support, and new therapeutic options, median survival rate has increased in patients with cystic fibrosis (CF) [1]. This condition has such a considerable effect that the number of adult patients with CF is now greater than the number of children in many developed countries [2,3]. Cystic fibrosis is a chronic and progressive disease that has an increasingly complex course in adulthood [4]. As a result of a chronic progressive illness, many disease- and treatment-related complications such as pulmonary exacerbations, massive hemoptysis, pneumothorax, cystic fibrosis-related diabetes, metabolic bone disease, adverse effects of medications, and psychological issues are more common in adulthood. However, respiratory failure is still the most common cause of mortality as observed in childhood population [5]. There are several methods such as spirometry, imaging, and lung clearance index to assess the severity of lung disease [6]. Spirometry is still the most commonly used method in registries for evaluating lung functions [2,3]. Determining the factors associated with severe lung disease may help to identify preventable and treatable risk factors associated with morbidity and mortality. There are no studies investigating risk factors related to poor lung capacity in Turkish adult population with CF as a developing country that has already established a registry system for CF [7].

With this study, as a newly established adult CF unit, we aimed to investigate clinical features of our adult population with CF and possible risk factors related with worse lung function.

## 2. Material and methods

### 2.1. Patients and methods

All patients over 18 years old with a confirmed diagnosis of CF (with a positive sweat test; sweat chloride ≥60 mmol/L) who were followed up in our adult CF unit between January 2014 and December 2015 were enrolled in this study. The data were collected during regular patient care. All patients gave informed consent for scientific use of the data. At the end of the enrollment period, a cross-sectional analysis was performed for investigating factors associated with severe lung disease. The demographic (age, sex), clinical and laboratory data (age at the time of diagnosis, the best value of body mass index (BMI) of the previous year, type of genetic mutation, sputum cultures, maximum value of forced expiratory volume in one second percent predicted (FEV1%) within the previous year, current comorbidities such as diabetes, pancreatic insufficiency, metabolic bone disease (osteopenia, osteoporosis), allergic bronchopulmonary aspergillosis (ABPA), the medical history (use of inhaled antibiotics, pancreatic enzyme replacement therapy, insulin, supplemental vitamins, pulmonary rehabilitation methods) and adverse reactions to drugs), number of hospitalization for intravenous antibiotic in the previous year were recorded. Genetic mutation analysis has been performed by sequence analysis since January 2015 in our hospital. Chrispin-Norman score was used for assessing the severity of lung disease using chest radiography [8]. Patients were divided into two groups based on FEV1% predicted value: group 1, severe lung disease with FEV1 <40%; group two, nonsevere lung disease with FEV1 ≥40%. These two groups were compared in terms of demographic, genetic, microbiological, laboratory features, comorbidity, medical therapy, and BMI. The patients were accepted as chronically infected with *Pseudomonas aeruginosa *or *Staphylococcus aureus *if these microorganisms were isolated in ³50% of routine sputum cultures during the study period [9]. Body mass index was categorized as <18.5, 18.5–22, 22–25, ³25 corresponding to underweight, risky, normal weight and overweight, respectively. Target BMI was accepted to be >22 kg/m2 for women and >23 kg/m2 for men [2]. Additionally, the median FEV1 value of the patients chronically infected with *Pseudomonas aeruginosa *and the patients with diabetes were compared with the patients who did not have these conditions. The study protocol was approved by the Ethics Committee at Hacettepe University (GO 15/146-39) and all participants provided informed consent in the format required by the relevant board.

### 2.2. Statistical analyses

We analyzed data with SPSS version 23.0.0.2 (SPSS, Chicago, Illinois, United States). We used mean and standard deviation for normally distributed continuous variables (age, BMI), median and minimum, maximum for nonnormally distributed data (age of diagnosis, FEV1, Chrispin-Norman score, hospitalization rate) and percentage for categorical variables. The Student’s *t*-test for independent samples was performed to compare means of age, and BMI between severe and nonsevere groups. Comparisons were made for continuous variables such as age of diagnosis, radiologic scores, hospitalization rates using the nonparametric Mann–Whitney test. Fisher’s exact test and chi-squared test were utilized for categorical comparisons of sex, comorbidity status such as diabetes, pancreatic insufficiency, presence of F508del mutation, chronic *P. aeruginosa* and *S. aureus* infection status, hospitalization at least once in a year, physiotherapy rate, and dornase alfa use. Additionally, median FEV1 values between patients with diabetes and nondiabetes were compared using the Mann–Whitney test. Correlations between FEV1 and BMI values were calculated using the Spearman correlation. 

## 3. Results

### 3.1. Patients

A total of 76 patients followed up in the study period were enrolled. Seventy-four patients (97.3%) were transferred from the department of pediatric pulmonology to adult CF unit. The mean age was 24.5 ± 5.25 years and male to female ratio was 1.1 (40/36 patients). The baseline characteristics of the patients are shown in Table 1. The median age at diagnosis was 96 (8.75–177) months and 22% were offspring of consanguineous marriage. Ten patients (13%) were diagnosed at adulthood.

**Table 1:** Demographic and clinical characteristics of patients.

	n = 76
Age (years); mean ± SD	24.5 ± 5.25
Female, n (%)	36 (47.4)
Age at diagnosis of CF (months);median (range)	96 (8.75–177)
Diagnosis over 18 years, n (%)	10 (13.1)
FEV1 % predicted; median (min, max)	64.5 (13, 121)
BMI (kg/m2), mean ± SD	21 ± 3.82
CFTR mutation (%)Class I-III homozygous	33
At least 1 Class IV-V mutation, n (%)	24
Unidentified/other, n (%)	43
F508del homozygous, n (%)	11 (14.4)
Chronic PsA infection, n (%)	23 (48.9)
Chronic S. aureus infection, n (%)	27 (57.4)
MRSA, n (%)	2 (2.6)
A. fumigatus, n (%)	18 (23.6)
Achromobacter spp., n (%)	7 (9.2)
S. maltophilia, n (%)	5 (6.5)
Nontuberculous mycobacteria, n (%)	4 (5.2)
Pancreatic insufficiency, n (%)	67 (88.2)
ABPA, n (%)	9 (11.8)
CFRD, n (%)	11 (14.5)
Metabolic bone disease, n (%)	27 (35.5)
Nasal polip and sinus disease, n (%)	18 (23.7) / 48 (63.2)
Liver disease, n (%)	28 (36.8)
Nephrolithiasis, n (%)	3 (3.9)
Pancreatitis, n (%)	2 (2.6)
Hemoptysis, n (%)	31 (40.8)
Pneumothorax, n (%)	4 (5.3)
Drug allergies, n (%)	13 (17.1)

n= number, SD= standard deviation, BMI= body mass index, FEV1= forced expiratory volume in 1 s, min= minimum, max= maximum, ABPA= allergic bronchopulmonary aspergillosis, CFRD= cystic fibrosis related diabetes, PsA= Pseudomonas aeruginosa, S. aureus= Staphylococcus aureus, MRSA= Meticilin resistant Staphylococcus aureus, A. fumigatus= Aspergillus fumigatus, S. maltophilia= Stenotrophomonas maltophilia, CFTR= cystic fibrosis transmembrane regulator

A total of 59 (77.6%) patients had genetic mutations. Of these, 39.4% were heterozygous for F508del and 14.4% were homozygous for F508del mutation. One-third of the patients were homozygous for a class I-III mutation. The median FEV1% predicted was 64.5 (13–121) and it was below 40 percentile in 18 (23.7%) patients. The mean BMI was 21 ± 3.82 kg/m2. Forty-eight (63.2%) patients had BMI below the targeted value and 18 (23.7%) patients were categorized as underweight. There was no patient receiving enteral nutrition support via nasogastric or gastrostomy tube. The medical history of the patients is summarized in Table 2. 

**Table 2:** Medical history.

	n (%)
Pancreatic enzyme replacement	67 (82)
Enteral nutrition support	23 (30.3)
Multivitamin	53 (69.7)
Dornase alpha	53 (69.7)
Bronchodilator	28 (36.8)
Inhaled steroid	22 (28.9)
Inhaled tobramycin	26 (34)
Inhaled colistin	16 (21)
Long term oxygen therapy	8 (10.5)
Oral steroid / itraconazol	7 (9.2)
Omalizumab	2 (2.7)
Insulin	11 (14.3)
Pulmonary rehabilitation (%)	
PEP-Flutter	30
Postural drainage	20
HCWO	3
None	47

n= number, PEP= positive end expiratory pressure, HCWO= high frequency chest wall oscillation

There was no lung transplant recipient in the study group, but 12 (15.8%) patients were on waiting list due to low FEV1% predicted (below 30%), recurrent severe hemoptysis or frequent pulmonary exacerbations. 

### 3.2. Severe lung disease and associated factors 

The median FEV1 values of females and males were 59.5 (14–121) and 67 (13–104), respectively (P = 0.8). The mean age of the severe lung disease group was higher than the nonsevere group (27.1 ± 6.0 vs 23.6 ± 4.7, p= 0.013). Three of ten patients diagnosed at adulthood had severe lung disease. There was no statistical difference in age of diagnosis between severe and nonsevere groups (102 months (5–384) vs 96 months (1–396)). The factors associated with worse lung function were shown in Table 3. There was a positive correlation between BMI and lung function, indicating that lower nutritional status was related to lower FEV1, r2 = 0.21, P < 0.001 (Figure). The median Chrispin-Norman score of severe lung disease group was higher than nonsevere group (14 (6–22) vs 5.5 (0–20), P < 0.001)*.* The proportion of patients with F508del mutation in at least one allele was similar to patients without F508del mutation in two groups (P = 0.46). The median FEV1% was lower in patients with CF-related diabetes (CFRD) (38 (14–95) vs 66 (13–121), P = 0.042). The median FEV1% of the patients colonized with P. aeruginosa was lower than others (56 (42–73) vs 68 (44–91), P = 0.17), but it did not reach a statistical significance. Dornase alpha use and physiotherapy rate were higher in severe lung disease group (P = 0.008 and P < 0.001, respectively). The median numbers of hospitalization for intravenous antibiotic therapy for pulmonary exacerbation in severe and nonsevere groups were 1 (0–12) vs 0 (0–3), respectively (P = 0.04). Hospitalization at least once in a year for intravenous antibiotic was more common in severe group (12/18 (66%) vs 19/58 (32%), P = 0.014). 

**Table 3:** Factors associated with severe lung disease.

	Group1: severe lung disease (FEV1 <40% predicted) ( n = 18)	Group 2: nonsevere lung disease (FEV1 ≥40% predicted) ( n = 58)	P-value
Female, n (%)	8 (44)	28 (48)	0.8
Age, mean±SD	27.1 ± 6.0	23.6 ± 4.7	0.013
Age of diagnosis, month, median (min-max)	102 (5–384)	96 (1–396)	0.4
BMI, mean±SD	18.5 ± 3.4	21.8 ± 3.6	0.001
CFRD, n (%)	6 (33)	5 (8)	0.009
Pancreatic insufficiency, n (%)	17 (94)	49 (84)	0.4
F508del at least one allel, n (%)	7 (38)	23 (39)	0.9
Chrispin norman score, median (min-max)	14 (6–22)	5.5 (0–20)	<0.001
Chronic P. aeruginosa, n (%)	5 (27)	18 (31)	0.7
Chronic MSSA, n (%)	5 (27)	22 (37)	0.4
Physiotherapy, n (%)	16 (88)	13 (22)	<0.001
Dornase alpha, n (%)	17 (94)	36 (62)	0.008
Hospitalization at least once in a year, n (%)	12 (66)	19 (32)	0.014

n= number, SD= standard deviation, BMI= body mass index, FEV1= forced expiratory volume in 1 s, min= minimum, max= maximum, CFRD= cystic fibrosis related diabetes, P. aeruginosa= Pseudomonas aeruginosa, MSSA= Meticilin-sensitive Staphylococcus aureus

**Figure:**
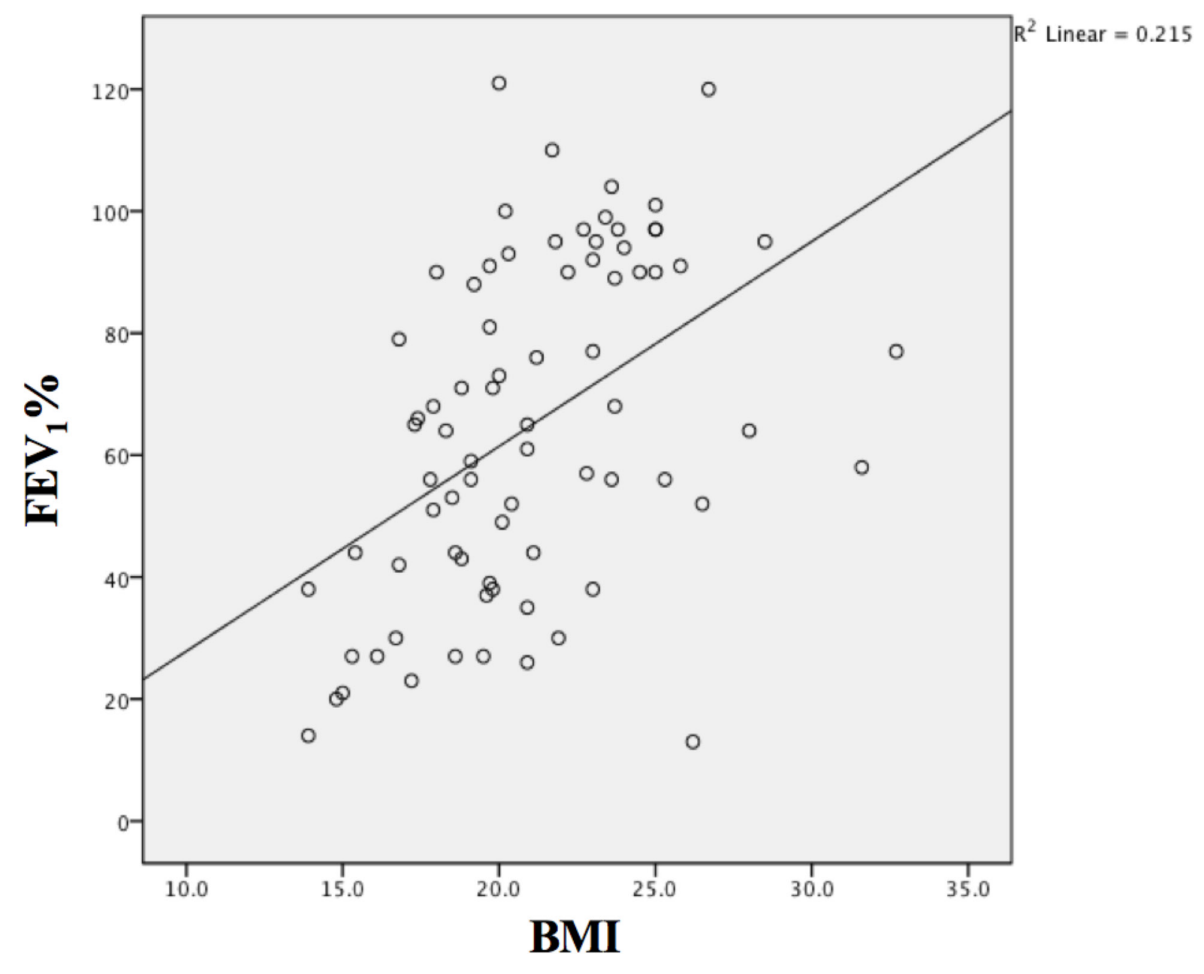
The correlation between forced expiratory volume in 1 s (FEV1%) and body mass index (BMI) values.

## 4. Discussion

To our knowledge, this is the first study evaluating risk factors related with worse lung function in an adult CF population in our country. We report that lower BMI, older age, presence of CF-related diabetes, higher Chrispin-Norman radiologic scores, use of dornase alpha and physiotherapy and higher hospitalization rate for intravenous antibiotic therapy are significantly associated with severe lung disease. 

Most of the patients were transferred from our pediatric department. The median age at the time of diagnosis was quite old (96 months) as compared with developed countries (4 months in US and 4.1 months in Europe) [2,3] and 10 patients were diagnosed during adulthood. The positive impact of early diagnosis on the course of the disease has already been known for years [10]. Therefore, the disease is now included in the neonatal screening program in many countries [11]. Turkey included CF in the neonatal screening program in 2015. We hope that early diagnosis with neonatal screening will reduce the number of patients diagnosed in adulthood. In our study, although the median age of diagnosis was not different between groups, the mean age of the patients with severe lung disease was higher. Longitudinal follow-up studies have showed FEV1 decline in the natural progression of the disease over years [12,13]. However, to discuss the relationship between advanced age and poor lung capacity, confounding factors should also be examined with larger cohort studies. Women were found to be colonized earlier with severe bacteria and had a decreased median life expectancy compared with men previously [14], but in our study, lung functions did not show difference between sexes. 

Most patients (63.2%) had BMI under the targeted value. There was an association between BMI and FEV1% predicted values. Many previous studies have emphasized the importance of nutritional support with a high calorie diet [15,16]. A study derived from European Cystic Fibrosis Society Patient Registry showed that patients with a lower BMI experienced a six-fold increased (95% CI 5.0–7.3) severe lung disease (FEV1% predicted <40) compared to patients with normal BMI [17]. Causality cannot be proven on cross-sectional data, as whether malnutrition is a cause or an effect of declining lung function discussed previously in a review, there is a circle of destruction consisting of lung damage from underlying disease, increased work of breathing, decreased ventilatory muscle mass, loss of respiratory muscles, and loss of weight. Poor nutrition is reported to lead to loss of muscle mass and to decreased diaphragm contractility [18]. On the other hand, according to a systematic review assessing nutritional intervention studies in cystic fibrosis, a significant difference in pulmonary function could not have been shown before and after nutritional interventions [19]. Although almost 25% of our patients were categorized as underweight, none of them accepted enteral nutrition support via nasogastric or gastrostomy tubes. We thought that lack of education and patients’ fear of the procedures could be the underlying reasons for this problem. 

Additionally, consistent with the literature, lower BMI and lower FEV1 were observed in CFRD group in our study. Diabetes is almost seen in 50% of CF patients in adulthood [20]. Its negative impact on disease progression and lung function is well-known; therefore, annual screening with oral glucose tolerance test (OGTT) is recommended since age of ten for CF patients [21]. In our CF unit, adult patients have been evaluated for CFRD by OGTT annually. Diabetes adversely affects disease prognosis through various mechanisms. In human diabetic lungs and in mouse models with CF as well, it was shown that pulmonary capillary blood volume, lung elastic recoil, and FEV1 are decreasing [22] in diabetes group. In a different study, computed tomography scans of diabetic patients were consistent with more significant airway thickening and parenchymal changes [23]. In addition, inspiratory muscle performance was found restricted in diabetic patients [24]. Moreover, airway glucose concentration’s effect on growth of respiratory pathogens was shown in a study in diabetic CF patients [25]. The majority of the study group (88%) had exocrine pancreas insufficiency. The association between worse lung function and pancreatic insufficiency was shown previously, but we could not detect a similar result when we compared the groups [26].

In our study, F508del mutation frequency was quite low compared to other European countries [3]. Cystic fibrosis transmembrane conductance regulator mutation distribution varies from country to country throughout the world [27]. Previously it was shown that patients with class 1-2 mutations had lower spirometric values and higher risk of developing severe lung disease [28]. Genotype is an important determinant of clinical prognosis, but there are also several risk factors which may affect clinical course of the disease [26]. We could not detect genotype–phenotype correlation in terms of lung functions in our patients. 

Chrispin-Norman score was used for evaluating chest radiographs, and the median value was higher in severe lung disease group as we expected. *Pseudomonas aeruginosa* and *S. aureus* were the most common pathogens isolated from the patients in our study. Chronic *P. aeruginosa* infection was shown to be associated with worse lung function and a major predictor of morbidity and mortality during both childhood and adulthood [29,30]. In our study, the frequency of chronic infection did not differ between the two groups. Prevalence of fungal infection with *Aspergillus *spp. was 23.6%. The clinical impact of fungi on lung functions is not clear yet although cross-sectional studies have shown negative effect on pulmonary function [31]. We observed patients infected/colonized with atypical pathogens such as *Stenotrophomonas maltophilia*, *Mycobacterium abscessus*, and* Achromobacter *spp., and their treatment and/or eradication of the pathogen was challenging. The relationship between these agents and lung function could not be investigated due to insufficient patient numbers in groups. 

Ninety-one percent of our patients with chronic *P. aeruginosa* infection received inhaled tobramycin or colistin. The beneficial effect of inhaled tobramycin and oral azithromycin on chronic *P. aeruginosa* infection was shown in several studies [32]. We could not detect a significant difference between the groups in terms of use of inhaled antibiotics. Dornase alfa was used as a mucolytic agent in 69.7%, and it was more prescribed in severe lung disease group. A study that compared lung functions of US and UK showed that hypertonic saline and dornase alfa were prescribed much more frequently and the patients had better lung functions in the US [33]. There are several ongoing trials about CFTR-modulator therapy in developed countries [34–36]. We could not use these drugs in our patients because modulator treatments are not covered by reimbursement yet.

Airway clearance techniques and chest physiotherapy are the cornerstones of CF treatment and it is recommended to all CF patients [37,38]. Although all patients were consulted to physiotherapy department in outpatient clinic, almost half of them were not performed regular chest physiotherapy. However, patients in the severe lung disease group were more compatible to physiotherapy in our study or they needed more physiotherapy. 

The median number of hospitalization was higher and hospitalization at least once in a year for intravenous antibiotic therapy for pulmonary exacerbation was more common in the severe group. It was previously shown that higher pulmonary exacerbation rates requiring hospital admission were associated with worse lung function [39]. 

Due to progressive lung disease, complications such as hemoptysis and pneumothorax are increasing in older ages [4]. The prevalence of massive or nonmassive hemoptysis was 40.8% and pneumothorax was 5.3% in our patient population (Table 1). Although CF is one of the most common lung transplantation indications worldwide, there was no transplanted patient in our clinic due to lack of sufficient experience in lung transplantation in CF in our country. However, 12 (15.8%) patients have been on waiting list. 

This is the first study focusing on the factors associated with severe lung disease in a Turkish adult population with CF. Although CF is a rare disease, as a reference center, we evaluated the largest adult patient data. As a newly established adult CF unit in our hospital, we collected the data regularly. As a limitation of our study, this data belongs to a single center and the patient number was not enough to evaluate the risk factors with a regression model. Hopefully, as the registry data increases over time, more information about adult patients will be provided. 

In conclusion, this study which includes the largest adult population with CF in our country demonstrates that lower BMI, older age, presence of CF-related diabetes, higher radiologic scores, use of dornase alpha and physiotherapy, and higher hospitalization rate for intravenous antibiotic therapy are significantly associated with severe lung disease. Respiratory failure is still the most common reason for morbidity and mortality; therefore, for preventing lung function decline, some factors like nutritional status of the patients, presence of CFRD and chronic infections should be seriously taken into account.

## Acknowledgments

The preliminary results of our clinic were presented at ECFS Conference, Basel, 2016. (P246: B. Er, N. Kiper, E. Celebioglu, U. Ozcelik, S. Emri. u2912). BE thanks Hacettepe University for providing grant opportunity (TBI-2015-5640, 2015) to visit Adult Cystic Fibrosis Unit at Harvard University for six months to gain experience about adult care of CF which helped to set up the infrastructure for this study.
